# Selecting Indicators to Monitor and Assess Environmental Health in a Portuguese Urban Setting: A Participatory Approach

**DOI:** 10.3390/ijerph17228597

**Published:** 2020-11-19

**Authors:** Marta Salgado, Ana C. L. Vieira, Anália Torres, Mónica D. Oliveira

**Affiliations:** 1 Institute of Environmental Health (ISAMB), Faculty of Medicine, University of Lisbon, 1649-028 Lisboa, Portugal; 2 Center for Management Studies of Instituto Superior Técnico (CEG-IST), Instituto Superior Técnico, Universidade de Lisboa, 1049-001 Lisboa, Portugal; ana.lopes.vieira@tecnico.ulisboa.pt (A.C.L.V.); monica.oliveira@tecnico.ulisboa.pt (M.D.O.); 3 Valorsul, Estação Mercadorias Bobadela, Plataforma Ribeirinha CP Lisboa, 2696-801 São João da Talha, Portugal; analia.torres@valorsul.pt

**Keywords:** environmental health indicators, urban settings, participatory approach, interview, Web-Delphi

## Abstract

Environmental health (EH) is influenced by complex interactions between health and the built and natural environments, there being little research on its specificities in urban settings. The use of suitable indicators to monitor and assess EH is fundamental in informing evidence-based interventions at the local level. A participatory approach to selecting indicators to inform the monitoring and assessment of EH in Lisbon is herein considered. Evidence derived from a systematic review of literature and data from Lisbon and Portuguese databases were analyzed by 12 Portuguese experts in individual semi-structured interviews. The interviews aimed at identifying relevant indicators and important emerging issues in the Lisbon urban setting. The outputs from the interviews were validated by a two-round Web-Delphi process in which panelists (22 experts) from different areas of expertise expressed their views regarding the relevance of the indicators for the analysis of EH in urban settings. Seventeen indicators were validated in the Web-Delphi process. High participation achieved along this process supports the view that this participatory approach was useful for validation. Results from the adopted participatory approach point out gaps in the collection of noise and mobility indicators data and raise emerging issues on housing indicators that require further research. The results also suggest the need for local action to improve indicators and tools in order to help the monitorization of EH in urban contexts. The adopted participatory approach can be replicated for other Portuguese and European urban settings.

## 1. Introduction

The United Nations 2030 Agenda for sustainable development goals (SDGs) recall the importance of placing at the top of the political agenda the pursuit of good health and wellbeing (SDG-3) and the promotion of sustainable cities and communities (SDG-11) [1]. Pursuing such goals requires the use of health information systems that enable the monitoring and assessing of health in multiple dimensions, with such systems entailing specificities across geographical settings [2,3]. Within such settings, it is recognized that health outcomes have a strong relationship with the built and natural environment, with this link becoming more evident in urban areas [4,5]. Although the disparities between urban–rural settings have been narrowing, residents in urban areas are more affected by physical factors [6,7]. As cities are home to more than 60% of the world’s population [5,8], the growing understanding of these relationships in the way cities are planned and built and how this impacts health has evolved over the years [9]. Nowadays, sustainable cities have come to the forefront of environmental health (EH) priorities [10].

The environmental health field comprises the monitorization and surveillance of built and natural determinants like air quality, noise, green spaces area, and walkability, and is concerned with the prevention of disease and with the creating of health-supportive environments [11,12]. One of the most demanding challenges for researchers and policymakers within the field is to isolate built and natural environment dimensions and their interactions and to be able to define an approach to managing these interactions, in order to create value. Associated with this challenge, comes the need to do so while collecting population’ intelligence through collaborative practices and to support innovation in EH interventions [13,14]. EH indicators are particularly important in monitoring and assessing the environment status and its trends in urban settings [15], in order to improve interventions, allocate resources, and investigate the effectiveness of health policies [16]. It is known that the collection of monitoring and surveillance data is a key task for many governmental organizations and industries charged with ensuring the wellbeing of the population [17]. Environmental interactions are difficult to quantify and evaluate, and to gather evidence-based information for decision-making is far from straightforward [10,18]. As it is essential to build evidence-based tools for the development of EH interventions, there must be a rationale on how to select the dimensions and indicators to assess EH [19]. Nevertheless, using this type of evidence to inform the design and evaluation of policies in urban settings is an under-researched area [20]. Mason and Lindberg [21] and Pine and Glonti [22] reported that researchers and policymakers value data and expertise in the local context. The literature also recalls that built and natural environment indicators must have a proven effect on health and that their selection should be based on a sound approach, so that they can become a resource in identifying potential risks to health [23].

Methods for selecting indicators vary from statistical and causal models [15] to systematic reviews of the literature [24,25] and participatory processes [26,27,28,29]. The use of participatory processes in EH research is especially important because of the complexity of the EH concept and of the interactions between EH determinants and health outcomes, as well as because of the importance of engaging experts with different knowledge in expressing their views and discussing local EH problems [30,31]. The perception of the complexity and specificities of urban settings also makes it crucial to consider a variety of experts’ views to increase the probability that the selected indicators will be considered credible, scientific, and relevant to EH interventions [32]. Multiple qualitative methods like interviews, surveys, or Delphi have been used for understanding complex issues related to EH analysis. Interviewing is a way of collecting data and can be used in various situations to cover a wide range of health topics [33]. Yet Brewerton [34] argues that interviews can have low reliability due to their openness to different types of bias. To overcome issues of the validity and reliability of the findings of an interview, other methods must be applied to serve as a guarantee of the participants’ performance. Another qualitative method widely used in health research is the Delphi process which captures experts’ opinion and can be used to promote consensus [35]. The Web-Delphi is being increasingly explored due to the use of technological platforms having the advantage of not requiring face to face contact, removing geographical barriers, and allowing the involvement of a large number of experts [36]. Delphi processes have been used to identify criteria to characterize and differentiate models of palliative care in healthcare services [27], to define core areas of EH [30], and to compare scenarios in order to improve urban sustainability development [37,38]. In this article, we propose the use of a Web-Delphi process as a tool to validate the information collected for a set of built and natural indicators.

Lisbon has been recently selected as European Green Capital 2020 with an ambitious agenda for the next decade, under the motto “Choose to evolve: measures for 2030” [39]. This agenda is fully aligned with the SDGs-2030 Agenda and implementing its goals requires setting out the data to be collected in the urban context, to monitor EH and to assess the extent to which goals are achieved. Accordingly, there is an opportunity and need to develop tools (for instance, based on dashboards and/or on composite indicators) that enable the monitorization and improvement of EH by local authorities and industries.

Thus, with a view to collecting key information for improving EH, this study explores an integrated participatory process to involve EH stakeholders and experts in the selection of the relevant dimensions and indicators from built and natural environment determinants, in order to monitor and assess EH in Lisbon, Portugal.

## 2. Materials and Methods

**Figure 1:**
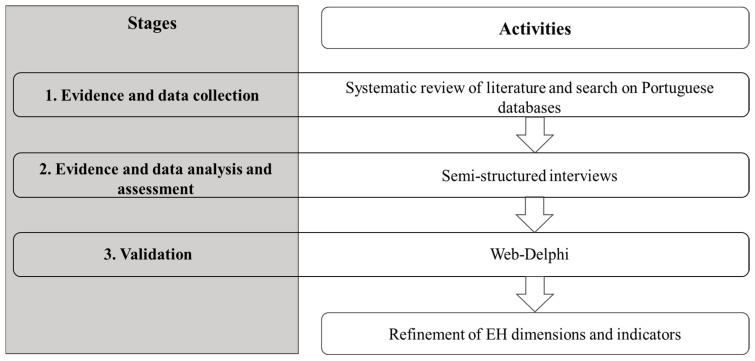
Overview of the approach used to select the key dimensions and indicators, from built and natural environment determinants, to evaluate environmental health (EH) in the Lisbon urban setting.

For the purpose of selecting relevant dimensions and indicators relevant for the evaluation of EH in Lisbon—informed by evidence, by data, and by the views of multiple EH stakeholders and experts—we designed a multi-methodology with three stages (see Figure 1 for an overview). The first stage comprises a systematic review of the literature that focuses on environmental determinants in urban settings and which is described in detail in Salgado et al. [20], along with a search and collection of data from Portuguese and Lisbon databases relevant to assess EH in urban settings. In the second stage, the outputs of the literature review were used as an input to semi-structured interviews. In the interviews, a restricted panel of Portuguese experts from relevant EH backgrounds made a detailed analysis and evaluation of the collected evidence and data and discussed which dimensions and indicators should be used to monitor and assess EH in Lisbon. Interviews were selected as they are powerful for eliciting narrative and complex data and allow researchers to investigate experts’ views in greater depth [33]. The third stage departed from the knowledge collected during the semi-structured interviews, and consisted of a two-round Web-Delphi process to promote a commitment among a larger panel of national experts towards consensus regarding the inclusion of the final set of dimensions and indicators in a tool to monitor and assess EH in Lisbon, as well as for collecting insights and issues related to these indicators. The Web-Delphi was used as a validation stage with the aim of providing complementary completeness, plausibility, credibility, and confirmation of the knowledge acquired in the semi-structured interviews [40], while enabling experts to interact in an anonymous format. Stages 1, 2 and 3 are detailed below.

### 2.1. Evidence and Data Collection: Systematic Review of Literature and Search on Portuguese Databases

The systematic review of literature reported in Salgado et al. [20] aimed at identifying the dimensions and indicators within the socioeconomic, built environment, natural environment, healthcare, and behavior determinants that impact on health in urban settings. For that purpose, the review used a search strategy based on the keywords “population health”, “city OR cities OR town OR “metropolitan area” OR “urban environment”, “indicators OR determinants”. The period covered in the search was from 2008 to 2018 and searches were performed in PubMed, Web of Science, Scopus and SciELO Portugal databases. Through screening of the literature, 94 studies were selected and included for analysis. The review detected case-control, cohorts, cross-sectional and ecological studies quantifying causal inferences that provided direct evidence to link an environmental risk factor, measured by the indicators, to a health outcome [41]. The results from the systematic review that focused on the physical determinants, i.e., built and natural environment, were summarized and used to build an initial list of potential dimensions and indicators relevant to evaluate EH in urban settings within the scope of this study.

A search in Portuguese national and local open-access databases, the Instituto Nacional de Estatística (National Institute of Statistics, INE, Portugal) [42], Lisbon city council [15], and PORDATA [43], was performed to collect data and analyze available information for each indicator proposed in the initial list, and to inquire about the spatio-temporal availability of these indicators for the city of Lisbon. Accordingly, a list of thirty-four indicators grouped into nine dimensions was gathered. The indicators and dimensions were organized according to the categories of built or natural environment. Along with the list of indicators, a description of each indicator was defined, together with information concerning geographical availability and measurement periodicity.

### 2.2. Evidence and Data Analysis and Assessment: Semi-Structured Interviews

Given the complexity of the evidence and the nature of data collected, face-to-face semi-structured interviews were conducted for the indicators and for data analysis and assessment. This method was selected for two reasons. First, the associated bidirectional communication between interviewer and interviewee helps in obtaining more detailed and in-depth information. Tacit in this reasoning is that interviewees cannot share a common vocabulary and some aspects might have to be clarified considering the different areas of expertise, improving the validity of the results [44,45]. Second, it allows the asking of other questions outside the semi-structured questionnaire to clarify data assessment and to ask follow-up questions. This flexibility in sharing knowledge was deemed appropriate for the context and helped generating unexpected insights.

Twelve semi-structured interviews were conducted with Portuguese experts working in Lisbon, chosen to represent equitably the variety of perspectives concerning EH: experts in built environment, natural environment, EH, and from national regulation institutions (see Table 1). Involved interviewees had to fulfill at least one of the following criteria: (1) more than five years of experience in EH-related research projects for the Lisbon area; (2) having publications related to air quality, urban sustainability, renewable energy consumption and production, influence of green areas on health, water quality; or (3) being superior technicians from national regulation institutions. The experts received a formal invitation via email along with a description of the study and the process of informed consent. The experts who consented to be interviewed received the questionnaire template in advance (see Supplementary Material) with the purpose of exploring the questions more systematically and comprehensively, as well as to keep the interview focused on the desired line of action. The questionnaire consisted of three parts. The first part collected interviewee’s analysis of the results on EH dimensions from the literature. The second part included questions related to the built and natural environment determinant indicators collected in national and local databases. The third part asked for opinions regarding missing information on indicators that should be included in order to evaluate EH.

In the first part, the interviewees rated their level of agreement with the inclusion of each dimension used to evaluate EH in Lisbon making use of a five-level Likert scale (SA: Strongly disagree, D: Disagree, N: Neither agree nor disagree, A: Agree, SA: Strongly agree), with an extra option of DNK/DNWA: do not know/do not want to answer. In the second part, the same rationale was followed for rating the level of agreement, with the inclusion of the indicators previously collected from the national databases. The interviewees were also asked to analyze the categorization of indicators within the dimensions, and to indicate which indicators could be seen as redundant, i.e., which could be considered as capturing the same phenomena. In the third part, the participants were invited to state if any indicators considered relevant to monitor and assess EH were missing, regardless of the availability of data for Lisbon, and if there was a possible strategy to measure those indicators. In the end, for each indicator for which data was only available at the national level, interviewees were asked whether the national data could be used as a proxy for local data. The answers were written by the interviewer on interview sheets. When processing the information gathered in all the interviews, the dimensions and indicators were considered relevant if more than 50% of the interviewees stated “strongly agree” or “agree” and, at the same time, did not have more than 25% of “strongly disagree” or “disagree” responses. Conversely, indicators with more than 75% of either “strongly disagree” or “disagree” were rejected. If any of the interviewees choose “DNK/DNWA” for any question, the answer was not considered for that dimension or indicator.

### 2.3. Validation: Web-Delphi Process

The use of the Web-Delphi method had a two-fold objective. Firstly, it aimed at gathering opinions of a wide range of experts with diverse backgrounds, and coming from various geographical locations, regarding the relevance of the indicators for assessing EH in urban settings, in order to validate the results from the interviews. Secondly, it exposed experts to alternative viewpoints from other experts, while allowing them to interact and reconsider their responses. The two-round Web-Delphi was conducted between February and March 2020.

#### 2.3.1. Delphi Panel

Evidence suggests that knowledge generation processes can be improved by ensuring diversity among the recruited participants [46]. For the purpose of this study, the Delphi panel members were defined as a group of members knowledgeable of and with work experience in the areas being studied and able to participate in the process [47]. The panel was chosen based on a “snowball” effect [48]. The interviewees were not included in the panel but recommended at least two other experts to participate in the Delphi process. The panel included a variety of expertise in air quality, epidemiology, environmental health, national institutions of health regulation, noise, public health, and urban sustainability. Based on these criteria, 55 Portuguese experts were invited to participate in the Web-Delphi. There is no general or specific rule for an optimal number of panelists, with this number varying in many Delphi studies [49,50,51]. In this study, the focus was on involving participants with heterogenous expertise and covering all relevant EH perspectives, as well as including experts from national and local institutions [52,53]. The main areas of expertise of Delphi participants are presented in Table 1.

#### 2.3.2. Web-Delphi Design

The information resulting from the interviews was taken as the input into the Web-Delphi process. If an indicator had been agreed for inclusion by a majority (more than 50%) of interviewees, that indicator was considered in the Web-Delphi rounds. The questionnaires were implemented via a web platform (available at www.welphi.com) [54]. As evidence suggests that a high number of rounds can result in a lower response rate [47,55], and Dalkey and Brown [56] notice that the best accuracy in response rates is obtained with two-round Delphi iterations with the drop-out increasing after two iterations, a two-round process design was adopted.

The process started with the online invitation of each panelist, containing instructions to access the Welphi platform, the informed consent, information regarding the study, and the first round questionnaire. The participants were asked to answer within ten workdays of receiving the email. Two reminders were sent to those participants who did not complete the survey. The Welphi platform was used to communicate with the panelists. Participants who did not complete the round during the deadline were not invited to the second round.

In the first round, panel members were asked to indicate their level of agreement or disagreement with each indicator on a five-level Likert scale (SD, D, N, A, SA), with the option of answering DNK/DNWA. Specific rules were applied to deal with differences in opinion and to measure the level of agreement. The rule adopted for the inclusion of an indicator, i.e., to have more than 50% of the panel members stating “strongly agree” and “agree” (agreement), and at the same time having less than 33% of answers rating as “strongly disagree” and “disagree” (SA+A > 50% and SD+D < 33.3%), were approved by an absolute majority. Additionally, an indicator receiving more than 75% of “SA” and “A” responses was then approved by qualified majority. Conversely, indicators with more than 50% of either “strongly disagree” or “disagree” responses were immediately rejected by absolute majority. In the second round, the rules for approval and rejection, as applied in the first round, were kept. The Web-Delphi questionnaire included the list of 17 indicators which resulted from the semi-structured interviews, along with information regarding the respective dimensions and spatio-temporal availability for Lisbon. The participants also had the opportunity to make comments and suggestions about each indicator in a comment section provided in the Welphi platform. In the second round, panel members had the opportunity to see the results from the first round (both participants’ choices and comments) and were invited to keep or revise their answers if they considered this appropriate.

#### 2.3.3. Statistical Analysis

The Web-Delphi process aimed at validating the results from the semi-structured interviews by promoting a higher level of agreement among the experts towards a consensus, there being nevertheless an expectation of divergence of opinion regarding either the inclusion or exclusion of some of the indicators. To understand such differences in opinion, Cronbach’s α was calculated to understand the extent to which there was alignment among all panelists. Cronbach’s α has been used previously as an association index to measure the level of agreement among panelists [57] and was used during each round of the Web-Delphi to estimate the association of the ratings in the unidimensional Likert scale [58]. The meaning of the value of Cronbach’s α depends on the context, but when Cronbach’s α is close to 1.0, it means that the participants’ choices are strongly associated, while values close to 0 suggest that the participants’ choices are highly unrelated to one another. A cutoff of the Cronbach’s α of 0.70 was adopted, taking the view that values above 0.7 are aligned with the inclusion of the indicator within an environmental health monitorization tool [16].

## 3. Results

### 3.1. Evidence and Data Collection: Systematic Review of Literature and Search of Portuguese Databases

Based upon the review of the literature, a summary of the 34 indicators related to built and natural environment determinants and with the potential to impact health in urban settings was selected (see Table 2). The selection was based on the causal inferences reported mainly in the cross-sectional and cohort studies included in the review of the literature, to ensure the validity of the evidence withdrawn. To complement this evidence, an additional search in national and local databases was performed to acquire spatio-temporal availability information (see Supplementary Material) for indicators measured in the Lisbon region and in Portugal. A description with details regarding the periodicity of measurement, geographical level and units of measure was made for each indicator (see Supplementary Material). The indicators for which information is currently available for the national level were identified, as well as the indicators lacking data. The evidence and data collection process identified five dimensions (green spaces, housing, mobility, safety, and sanitation) 18 indicators from the built environment; and four dimensions (air quality, noise, soil, and water quality) and 16 indicators for the natural environment. The indicators were grouped into these dimensions and an initial list of nine potential dimensions and 34 indicators was proposed for analysis in the next stage (see Supplementary Material and Table 2).

### 3.2. Evidence and Data Analysis and Assessment: Semi-Structured Interviews

The 12 semi-structured interviews were performed with experts whose main expertise related to the built environment, natural environment, EH, and national health regulation institutions, as shown in Table 1.

Each interview took a median of 55 min (range 45–70 min). The first part of the interview gathered views from participants regarding EH dimensions to be used to monitor and assess EH in Lisbon. From the set of nine dimensions, six dimensions reached a high level of agreement—more than a 50% agreement rate—about their relevance in evaluating EH in Lisbon. Over half of the interviewees disagreed with the inclusion of the safety and sanitation dimensions. Soil dimension was indicated to be important in evaluating EH in rural settings, but for cities, it was not seen as relevant for inclusion.

In the second part, the interviewees stated their agreement regarding the indicators collected in the national and local databases search. An early finding at this stage was that experts were very concerned about the availability of the data when stating their agreement regarding the inclusion and/or exclusion of the indicators, even if they had been instructed to consider indicators for which data might not exist. For some indicators, the interviewees chose not to state their opinion (DNK/DNWA). From the initial list of 34 indicators, a total of 16 (47%) were considered as relevant for monitoring and assessing EH in Lisbon by the majority of the interviewees. The indicator “Public water supply” in the “Water quality” dimension was considered as important only for cities affected by drought episodes, which is not the case for Lisbon. The “Build-up area” indicator was considered redundant in the light of other indicators included in the “Green spaces” and “Mobility” dimensions. The “Protected area surface”, “Protected area proportion” and “Number of gardens and parks” indicators were also considered redundant. Indicators measuring the area of leisure parks and community gardens achieved a higher agreement than the indicators considered redundant (see Table 2).

The last part of the interview gathered opinions regarding missing information that should be included to monitor and assess EH, with six interviewees proposing an additional indicator— “energy poverty vulnerability index”—for the housing dimension.

Based on the evidence and information collected during the semi-structured interviews, a final set of indicators and dimensions was organized. Hence the built environment determinant was divided into three dimensions—green spaces, housing, and mobility- and the natural environment determinant into another three dimensions—air quality, noise, and water quality. These six dimensions encompassed a total of 17 indicators, six in the built environment determinants, and 11 in the natural environment determinants. This set of dimensions and indicators was the input for the Web-Delphi process in the validation stage.

### 3.3. Validation: Web-Delphi Process

#### 3.3.1. Panel Participation

From the 55 experts invited for the Web-Delphi process, 29 accepted the invitation and completed the first round of the Web-Delphi questionnaire (53% response rate). In the second round, 22 experts completed the questionnaire (76% response rate). A high response rate was achieved from the built environment experts where all participants completed both rounds. The group of respondents in the final round had a balanced number of experts from all areas of expertise (see Table 1).

#### 3.3.2. Indicators

A list of 17 indicators, grouped in six dimensions, resulted from the interviews and was validated as relevant in monitoring and assessing EH in Lisbon city, as captured by the high agreement rate among experts in the Web-Delphi. In summary, ten indicators were validated by absolute majority (SA > 50% and SD+D < 33.3%): nine from the natural environment determinant and one from the green spaces dimension (“Area of leisure parks and gardens”). Seven indicators were validated by qualified majority (SA+A > 50%): five from the built environment and two from the natural environment. The five indicators from the built environment included “Area of community gardens” from the green spaces dimension, and four indicators in the housing and mobility dimensions. The two indicators included from the natural environment were the SO_2_ and L_den_ indicators. Results show that the larger group validated the work taken from the evidence and data collection and from the interviews. The “Energy poverty vulnerability index” and the “Água segura” indicators reached the highest level of agreement (Table 3).

#### 3.3.3. Group Agreement Analysis

Analyzing the Cronbach’s α index results and the distribution of the group’s responses along the two rounds of the Web-Delphi process, it is possible to understand if the heterogeneity of Delphi panel members (in terms of expertise) translated into variability in Delphi answers in each indicator. Cronbach’s α for the first round of the Web-Delphi process was 0.80, indicating substantial agreement among the panelists’ answers from the beginning of the process. 22 experts completed the second round, having the opportunity to see the results from the first round and to revise the answers, with Cronbach’s α increasing from 0.80 to 0.84. An alpha higher than the established reliability cutoff of 0.70 indicates that, despite the different areas of knowledge from experts, they had similar views regarding the relevance of the indicators to monitor and assess EH in Lisbon.

## 4. Discussion

Our results contribute to deepening the knowledge of which indicators are relevant to monitor and assess EH in urban settings, and answered the need, identified in other studies, for the development of new approaches to selecting indicators to monitor and assess EH and to improving collection of EH-related data [59]. This is, to the best of our knowledge, one of the first studies attempting to combine empirical evidence, collected data, and experts’ views in the selection of dimensions and indicators to monitor and assess EH in a city in a holistic way, taking the view that the selection of indicators is at the cornerstone of the monitorization and assessment of EH [60].

### 4.1. Methods

The proposed multi-methodology was designed to help in structuring a transparent and tendentially more informed set of indicators useful in analyzing EH in the Lisbon urban area. The combination of interviews and Delphi processes was successful in promoting agreement on a set of indicators.

In this study, the level of agreement was used instead of consensus because it is easily interpretable, consensus being a specific case of agreement (perfect agreement) [61]. To our knowledge there are few studies in which the Delphi method using a web-platform was employed as a validation tool. The high level of agreement achieved in the two rounds proved sufficient to validate the inclusion of the indicators obtained from a smaller group of experts in a set of semi-structured interviews. The opportunity given in the Web-Delphi process to the experts to change their opinion, as a result of considering the views of their peers, was revealed to be a strength of the process. Cronbach’s α association index higher than 0.80 indicates that, besides having different areas of expertise, the experts appeared to have a shared knowledge of which indicators should be included to assess EH in Lisbon, and they were aware of the need to monitor EH with suitable indicators in the urban setting context.

### 4.2. EH in Urban Settings

Previous research has shown that EH has distinct patterns across urban and rural settings [62], with the results of our study providing evidence aligned with the literature. In rural settings, water and sanitation dimensions have been shown to have more impact on health [63], and Badland et al. [25] showed that air pollution, housing, public open spaces, and transport are the most important aspects in assessing health in urban settings. The set of green spaces, housing, mobility, air and water quality, and noise indicators validated in our study corroborate these findings, as well as revealing information gaps in data collected for the indicators used to analyze EH within the built and natural environment dimensions.

Although there is a lack of data for some built environment indicators in Lisbon, a high level of agreement was achieved on which of these indicators were relevant in monitoring EH. Out of the six indicators validated in the Web-Delphi for built environment dimensions, the “Area of community gardens” indicator was the one with the lowest agreement. Home and Vieli [64] state that urban agriculture performed in community gardens can meet some of the basic nutrition needs of the urban population and potentially provide economic, social, and mental and wellbeing benefits. During the interviews, the advantage of including this indicator was clear (83% agreement rate) while in the Web-Delphi this indicator achieved a rate of agreement of 55%. The difference in the results could be explained by the controversy around the importance of this type of green space when looked at alongside the availability of parks and gardens. The agreement attained towards the inclusion of the “Road network” and “Number of road vehicles” indicators can be further associated with the recognition of their impact on air and noise pollution [37,65]. In fact, to improve EH in the Lisbon context, some interventions have tried to control the number of cars in the city and to increase the number of cycling roads [37,39,65]. The surprising result from our participatory approach was the inclusion of the energy poverty vulnerability indicator in the housing dimension. Despite the need for data collection, this result corroborates that energy poverty vulnerability is a growing societal challenge that puts the welfare of the population at risk. The access to suitable housing conditions is one of the SDGs targets, and our results are in agreement with the idea that in Portugal there is growing awareness and research on the housing conditions dimension [66].

As opposed to the lack of information regarding built environment indicators, a larger number of natural environment indicators is regularly measured in Portugal. Air and water quality indicators are publicly available, updated, and achieved a high rate of agreement in the Web-Delphi. From the air quality indicators, the SO_2_ indicator was the only one included by qualified majority (an absolute majority was observed for the other air quality indicators). In fact, SO_2_ is one of the most important pollutants in the atmosphere that contributes to the degradation of buildings, and the main source is industry; exposure to high SO_2_ concentrations can lead to cardio-respiratory mortality and morbidity [67]. However, one should note that Lisbon is not an industrialized city, which could possibly have influenced the results achieved in the Web-Delphi. Another dimension validated in our process was noise. The causality of the effect of noise on health has not been sufficiently elucidated, and this dimension is often missing from frameworks to assess health [68]. However, the results of our study show that its potential impact on health should not be neglected, and measures of sound level should be up to date and tools designed to monitor EH in urban contexts. Overall, our results regarding the natural environment are aligned with the evidence showing air quality and access to good quality water as major concerns to urban residents [69] and point out the importance of including the noise dimension.

Many policies have been adopted in Lisbon assuming the interconnectivity between the built and the natural environments. For instance, interventions in mobility, such as reducing cars in the city and improving shared commuting, have been adopted for reducing the negative impact of air and noise pollution on health [3,39]. Another example is the awareness of improving access to green spaces, which also recognizes the need to pay proper attention to soil-related indicators like the presence of unsealed soils [70]. In our study, soil did not reach the level of agreement to be included as relevant to assess EH in Lisbon. This may potentially be explained by the fact that soil is generally taken as relevant for health in rural settings and perceived as a mere supporting platform in urban areas. Nevertheless, the literature has pointed out that urban soils can improve the wellbeing of residents and mitigate the effects of the current climate crises, and it may be necessary to start collecting data for these indicators at local level [71].

Following the findings of our study, it is clear that there is a need for gathering diverse and robust evidence to be further used to inform not only the monitoring of EH in urban settings but also the design and analysis of the impact of EH policies. The list of indicators that resulted from this study within SDGs and within the scope of an European Green City can be a starting point in identifying entry points for planning interventions targeting increased EH in Lisbon; and the set of indicators and dimensions could be the basis to discuss indicators and data collection in other European urban settings and the development of EH related information systems. The next stage of our work could involve structuring the set of selected and validated indicators into tools via which the EH improvements could be assessed and monitored (for instance, within a dashboard or within a EH index for urban settings).

### 4.3. Strengths and Limitations 

The complex and multisectoral nature of EH required a multi-disciplinary approach to develop a robust and comprehensive set of key dimensions and indicators to improve health in the Lisbon context. The adopted approach guaranteed that indicators from different areas of concern were canvassed in the final recommendations. By requiring that the initial set of indicators was filtered, analyzed, discussed, and validated through the experience of Portuguese experts, this study also serves as a test for the adequacy of the selected indicators in monitoring EH in the Lisbon city, and a template for comparison with other cities. The Web-Delphi process is structured and transparent and has shown to be useful as a validation tool. In addition, the use of a web-platform to build and deliver the questionnaire and to follow-up the process increased its efficiency. To address the potential bias of the Delphi studies, feedback was provided in an impartial way without influencing the responses. The anonymity of the experts was also assured.

Despite these aspects, this study design has several limitations. Semi-structured interviews allow the interviewer to discuss the questionnaire, but may be subject to bias, although the researcher was aware of the need to stay open to the suggestions of the interviewees. A further limitation of the study was the method used to identify the experts for the semi-structured interviews and for the Web-Delphi. While the recruitment protocol aimed to engage a multidisciplinary sample of experts, it should be acknowledged that a degree of convenience sampling was unavoidable and could have been tackled with a broader search to identify experts. Other limitations include the lack of guidance on Cronbach’s α thresholds, with 0.70 being adopted as the level after which there was a concordance between experts. Given the complexity of the EH concept, the results of this study could be improved if a higher number of experts were involved.

## 5. Conclusions

Aligned with the SDGs and with the current labelling of Lisbon as the European Green Capital 2020, this study led to the identification of six dimensions and 17 indicators essential to monitor EH in Lisbon city. A key finding is that a lack of data precludes the use of relevant indicators to monitor and assess EH in the Lisbon urban context. It is common that indicators are insufficiently tracked, and that many researchers and policymakers favor using fewer indicators with already collected data, rather than compiling a list of relevant indicators and then pushing for data collection. The adopted participatory approach highlights the usefulness of involving relevant experts from key fields of expertise in the selection of dimensions/indicators regarding EH; and the combined use of evidence, interviews and of a Web-Delphi process providing a consensual list of key indicators has shown to be transparent and flexible for replication in other EH contexts. The work developed in this study can inform the construction of tools to monitor EH, as well as helping policymakers in the definition of improvement goals and also in monitoring the extent to which those goals are fulfilled.

## Figures and Tables

**Table 1:** Profile of the participants in stages 2 and 3 of the approach used to select the key dimensions and indicators to evaluate EH in the Lisbon urban setting.

	Stage 2	Stage 3	
Item	Interviews	1st Round Web-Delphi	2nd Round Web-Delphi
Gender, *n* (%)			
Female	6 (50%)	14 (48%)	10 (45%)
Male	6 (50%)	15 (51%)	12 (55%)
Main area of expertise, *n* (%)			
Built environment	3 (25%)	5 (17%)	5 (23%)
Natural environment	3 (25%)	9 (31%)	5 (23%)
Environmental health	3 (25%)	10 (35%)	8 (36%)
National health regulation institutions	3 (25%)	5 (17%)	4 (18%)

**Table 2:** List of the 34 indicators retrieved from the literature and from databases search, and synthesis of experts’ opinions collected in the semi-structured interviews regarding the relevance of indicators in monitoring and assessing EH in the Lisbon urban setting.

Built Environment Determinant				
Dimension	Indicator	Disagreement (%)	Agreement (%)	Included
Green Spaces	Number of zoos, botanic parks, and aquariums *	75.0	0	No
	Protected area surface (km^2^)	83.4	0	No
	Protected area proportion (km^2^)	75.0	0	No
	Number of gardens and parks	75.0	0	No
	Area of leisure parks and gardens (m^2^)	0	83.3	Yes
	Area of community gardens (m^2^)	0	83.3	Yes
Housing	Number of classic family housing buildings *	83.3	0	No
	Number of buildings by geographic location and type *	75.0	0	No
Mobility	Built-up areas (km^2^) *	66.7	0	No
	Road vehicles by type and fuel *	16.7	58.4	Yes
	Traffic accidents with victims/1000 inhabitants	66.7	0	No
	Cycle roads (km)	0	75.0	Yes
	Road network (km)	0	75.0	Yes
	Proportion of resident population using individual mode of transport while commuting *	66.7	0	No
	Proportion of resident population using public transport mode during commuting *	58.3	0	No
Safety	Police-reported crimes/1000 inhabitants *	58.3	0	No
Sanitation	Urban waste collection *	58.3	0	No
	Population served by wastewater drainage systems *	58.3	0	No
**Natural Environment Determinant**				
Air quality	PM_2.5_ (particulate matter)	0	83.3	Yes
	PM_10_ (particulate matter)	0	83.3	Yes
	O_3_ (ozone)	0	83.3	Yes
	NO_2_ (nitrogen dioxide)	0	83.3	Yes
	NO_x_ (nitrogen oxides)	8.33	75.0	Yes
	SO_2_ (sulfur dioxide)	0	83.3	Yes
	CO (carbon monoxide)	0	83.3	Yes
	C_6_H_6_ (benzene)	8.33	75.0	Yes
Noise	Exposure to high noise	75.0	16.7	No
	Suffering from noise *	66.7	25.0	No
	L_den_ (day-evening-night equivalent sound level)	0	83.3	Yes
	L_n_ (day-night equivalent sound level)	0	83.3	Yes
Water quality	“Água segura” *^,a^	0	91.7	Yes
	Public water supply *	58.3	0	No
Soil	Soil organic carbon content *^,b^	100	0	No
	Soil sealing index *^,c^	75.0	16.7	No

Legend: * indicators for which information is currently available only for the national level; a: “Água segura” is the Portuguese indicator that measures the percentage of compliance with the parametric values established in the legislation; b: amount of organic carbon capable of affecting groundwater; c: percentage of unsealed soil.

**Table 3:** Indicators approved by majority decision rules after the second round, grouped by built and natural environment determinants.

Determinant	Dimension	Indicators	Agreement Rate (%)	Inclusion By
Built environment	Green spaces	Area of leisure parks and gardens	63.6	Absolute
		Area of community gardens	54.5	Qualified
	Housing	Energy poverty vulnerability index	95.5	Qualified
	Mobility	Cycle roads	86.4	Qualified
		Number of road vehicles	81.8	Qualified
		Road network	77.3	Qualified
Natural environment	Air quality	PM_2.5_	86.4	Absolute
		PM_10_	63.6	Absolute
		O_3_	63.6	Absolute
		NO_2_	63.6	Absolute
		NO_x_	59.1	Absolute
		SO_2_	86.4	Qualified
		CO	59.1	Absolute
		C_6_H_6_	54.5	Absolute
	Noise	L_den_	90.9	Qualified
		L_n_	63.6	Absolute
	Water quality	“Água segura”	95.5	Absolute

## Supplementary Materials

The following are available online at https://www.mdpi.com/1660-4601/17/22/8597/s1, Interview template.
