# Supplementary material for: Selecting Indicators to Monitor and Assess Environmental Health in a Portuguese Urban Setting: A Participatory Approach
Source: Int J Environ Res Public Health. 2020 Nov 19;17(22):8597. doi: 10.3390/ijerph17228597 (PMC7699361; doi:10.3390/ijerph17228597)
Supplement: Supplementary file 1 [file ijerph-17-08597-s001.pdf]

Interview template

This semi-structured interview will be performed to discuss the results obtained in a systematic review of literature and in a search to collect indicators in databases in Portugal, in view of selecting dimensions and indicators relevant to include in a framework for monitorization and evaluation of environmental health in urban settings, with focus on Lisbon.

This interview is structured in three parts. When answering to the questions, please bear in mind that the focus is the monitorization and evaluation of environmental health in Lisbon, but answers should not be constrained by the lack of data.

PART 1- Analysis of evidence from the literature review

Discussing the results on environmental health dimensions from the literature

The following dimensions have been found to be determinants of environmental health in the literature:

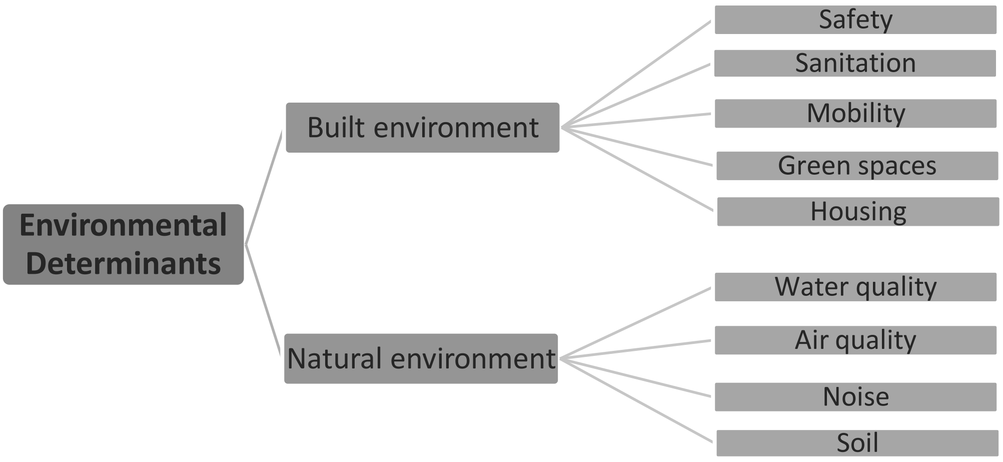

Figure 1 Environmental health dimensions

a) Do you agree that all the relevant dimensions in the natural and built environment are included in a framework (Figure 1) for monitoring and evaluating environmental health in Lisbon? Do you have suggestions of extra dimensions?

b) Within the natural environment component, past frameworks included **Soil** as a dimension to consider, but no evidence on association between soil and environmental health was found. Do you agree with the exclusion of Soil dimension to evaluate health in urban settings? If NO, why?

c) Within the built environment component, no evidence of association was found in the review between **Sanitation and Housing** indicators and health outcomes. Do you agree with the exclusion of these dimensions to evaluate health in urban settings? If NO, why?

PART 2- Analysis of indicators and data collected from Portuguese databases and of indicators' availability

1- Discussing on NATURAL ENVIRONMENT determinants' indicators collected for Portugal and/or Lisbon

Having in mind the aim to build a framework to be applied to Lisbon, a list of available indicators from national databases were collected. We grouped those indicators within dimensions previously reported in literature.

Please see the overview of indicators and dimensions before answering the questions.

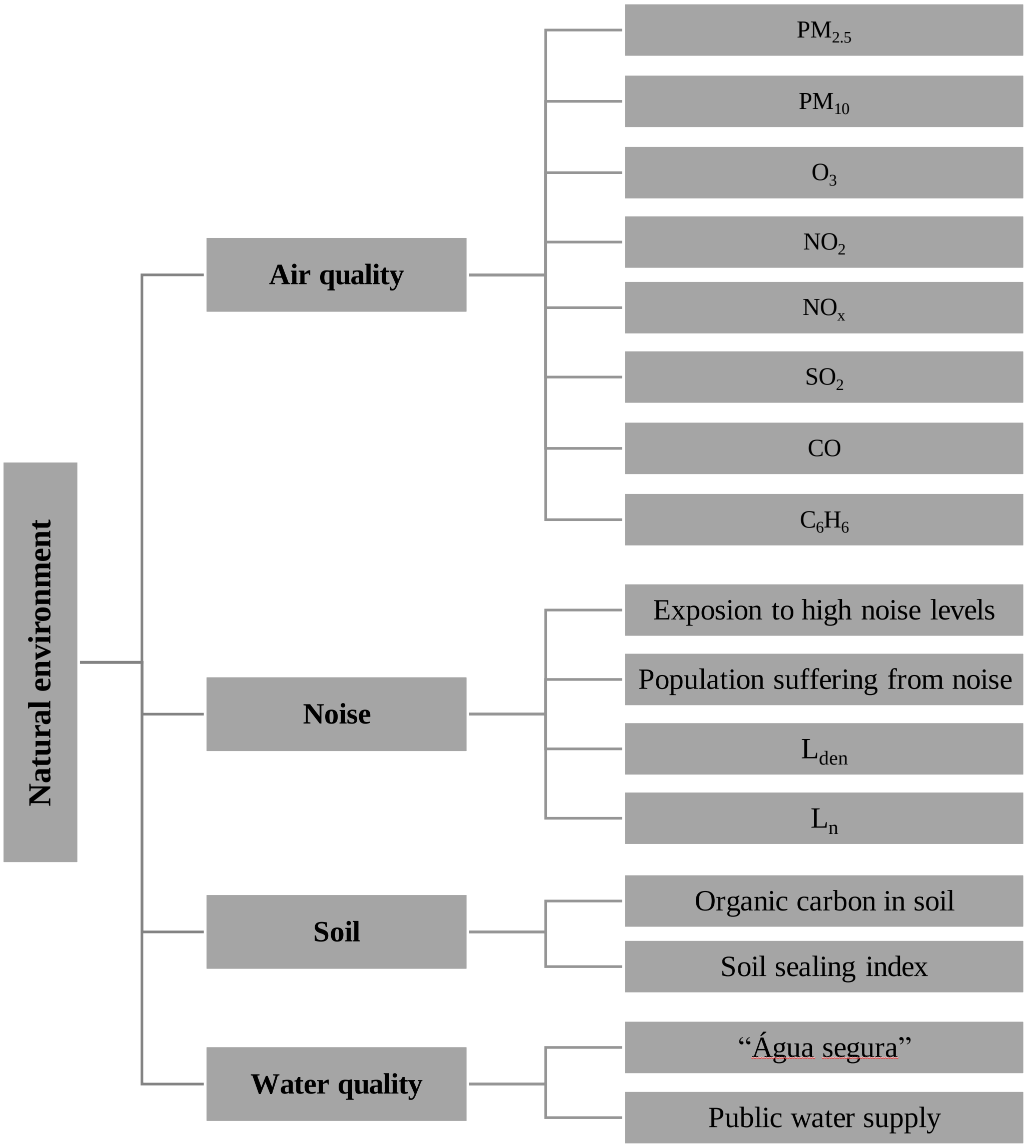

a) Please indicate the extent to which this indicator is relevant to monitor and evaluate environmental health in Lisbon:

Indicator	Strongly disagree	Disagree	Neither agree nor disagree	Agree	Totally Agree	DNK/ DNWA
$PM_{2.5}$						
PM_{10}						
O_3						
NO_2						
NO_X						
SO_2						
CO						
C_6H_6						
Exposure to high noise						
Suffering from noise						
L_{den}						

L_n						
Organic soil in soil						
Soil sealing index						
“Água segura”						
Public supply						

(DNK/DNWA: Do Not Know/Do Not Want to Answer**)**

b) Please indicate if each of the following indicators are included in the correct dimension:

Indicator	Dimension	If you disagree with the inclusion of the indicator in this dimension, describe which would be the correct dimension
$PM_{2.5}$	Air quality	
PM_{10}		
O_3		
NO_2		
NO_X		
SO_2		
CO		
C_6H_6		
Exposure to high noise	Noise	
Suffering from noise		
L_{den}		
L_n		
Organic soil in soil	Soil	
Soil sealing index		
“Água segura”	Water quality	
Public supply		

c) Please make a cross on the indicators that may be seen as redundant (i.e. which capture the same phenomena):

	$PM_{2.5}$	PM_{10}	O_3	NO_2	NO_X	SO_2	CO	C_6H_6	Exposure to noise	Suffering from noise	L_{den}	L_n	Organic soil in soil	Soil sealing index	“Água segura”	Public supply
$PM_{2.5}$																
PM_{10}																
O_3																
NO_2																
NO_X																
SO_2																
CO																
C_6H_6																
Exposure to high noise																
Suffering from noise																
L_{den}																
L_n																
Organic soil in soil																
Soil sealing index																
“Água segura”																
Public supply																

2- Discussing the results on BUILT ENVIRONMENT determinants' indicators collected for Portugal and/or Lisbon

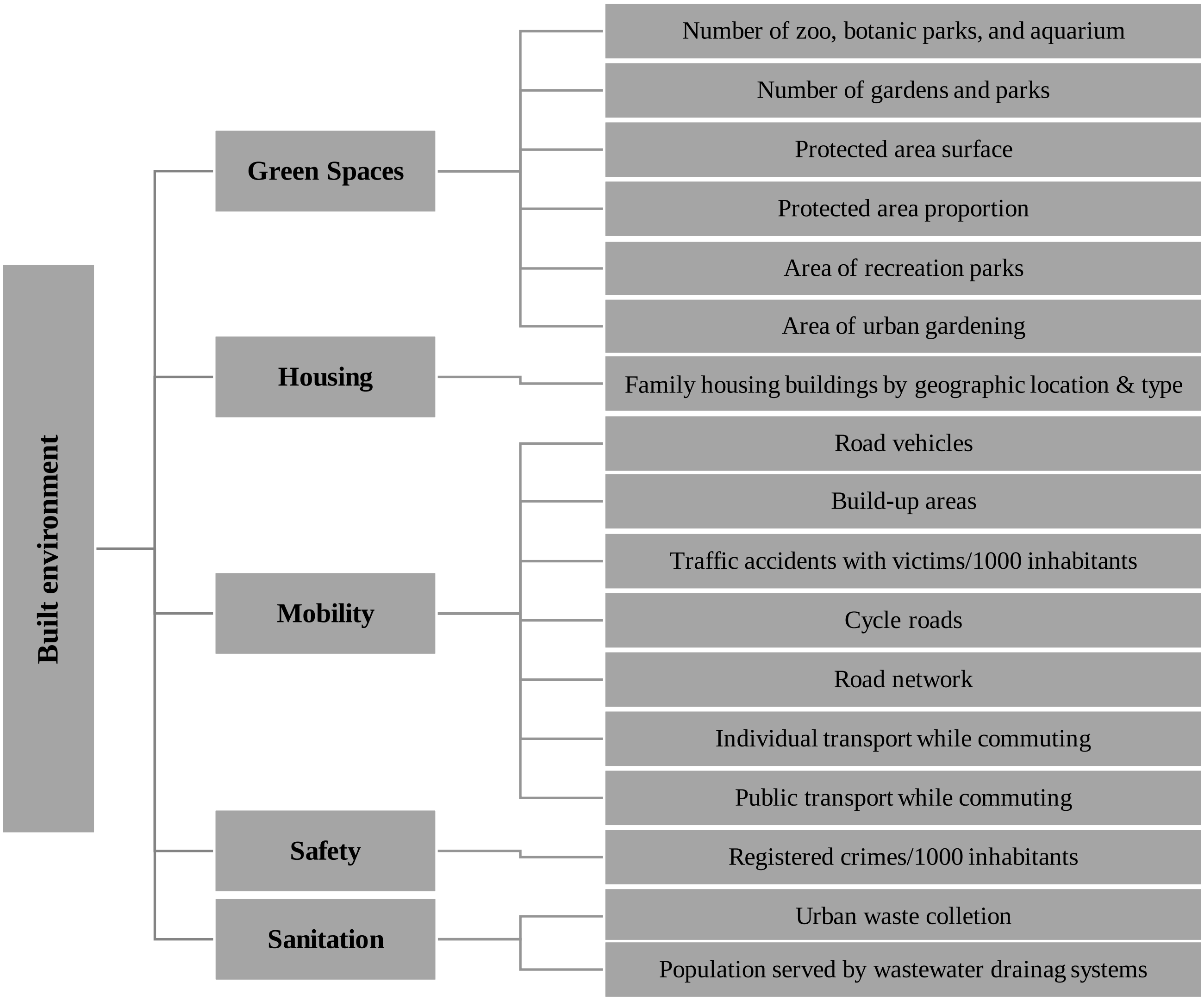

a) Please indicate the extent to which this indicator is relevant to monitor and evaluate environmental health in Lisbon:

**Indicator**	**Strongly disagree**	**Disagree**	**Neither agree nor disagree**	**Agree**	**Totally Agree**	**DNK/ DNWA**
Nº Zoo, botanic parks, and aquarium						
Protected area surface						
Protected area proportion						
Number of gardens and parks						
Area of recreation parks						
Area of urban gardening						
Classic family housing buildings						
Buildings by geographic location and type						
Built-up areas						
Road vehicles by type and fuel						
Traffic accidents with victims/ 1000 inhabitants						
Cycle roads						
Road Network						
Proportion of employed or student resident population using individual mode of transport while commuting						
Proportion of employed or student resident population using public transport mode during commuting						
Police-reported crimes /1000 inhabitants						
Urban waste collection						
Population served by wastewater drainage systems						

b) Please indicate if each of the following indicators are included in the correct dimension:

Indicator	Dimension	If you disagree with the inclusion of the indicator in this dimension, describe which would be the correct dimension
Nº Zoo, botanic parks, and aquariums	Green spaces	
Protected area surface		
Protected area proportion		
Number of gardens and parks		
Area of recreation parks		
Area of urban gardening		
Classic family housing buildings	Housing	
Buildings by geographic location and type		
Built-up areas	Mobility	
Road vehicles by type and fuel		
Traffic accidents with victims/ 1000 inhabitants		
Cycle roads		
Road Network		
Proportion of employed or student resident population using individual mode of transport while commuting		
Proportion of employed or student resident population using public transport mode during commuting		
Police-reported crimes /1000 inhabitants	Safety	
Urban waste collection	Sanitation	
Population served by wastewater drainage systems		

c) Please make a cross on the indicators that may be seen as redundant (i.e. which capture the same phenomena):

		1	2	3	4	5	6	7	8	9	10	11	12	13	14	15	16	17	18
1	Nº Zoo, botanic parks, and Aquarius																		
2	Protected area surface																		
3	Protected area proportion																		
4	Number of gardens and parks																		
5	Area of recreation Parks																		
6	Area of urban gardening																		
7	Classic family housing buildings																		
8	Buildings by geographic location and type																		
9	Built-up areas																		
10	Road vehicles by type and fuel																		
11	Traffic accidents with victims/ 1000 inhabitants																		

12	Cycle roads																		
13	Road Network																		
14	Proportion of employed or student resident population using individual mode of transport while commuting																		
15	Proportion of employed or student resident population using public transport mode during commuting																		
16	Police-reported crimes /1000 inhabitants																		
17	Urban waste collection																		
18	Population served by wastewater drainage systems																		

PART 3- Analysis of missing data

1- After analyzing above the available indicators, in your opinion, **which indicators are missing** to adequately monitor and evaluate environmental health in Lisbon?

a) Please indicate your suggestions of what should be included even if you do not know if data is available.

Determinant	Dimension	Possible indicator
**Built environment**	Green spaces	
	Housing	
	Mobility	
	Safety	
	Sanitation	
**Natural environment**	Air quality	
	Noise	
	Soil	
	Water quality	

b) Can you suggest a possible strategy to find information on missing dimensions/indicators?

2- The indicators presented in the tables below are indicators measured at national level. Please indicate if the national indicator can be used as a proxy for the urban level in Lisbon?

**Indicator**	**Periodicity**	**Year**	**Proxy for urban level?**
"Água segura"	Annual	2017	
Access to public water supply	Annual	2016	
Population living in households considering that they suffer from noise, by poverty status	Annual	2018	
Organic carbon in soil		2010	
Soil sealing index	3 years	2015	

**Indicators**	**Periodicity**	**Year**	**Proxy for urban level?**
Buildings by geographic location and type	Ten-year	2011	
Built-up areas	3 years	2015	
Classic family housing buildings	Annual	2018	
Nº Zoo, botanic parks, and Aquarius	Annual	2017	
Proportion of employed or student resident population using individual mode of transport while commuting	Ten-year	2011	
Proportion of employed or student resident population using public transport mode during commuting	Ten-year	2011	
Road vehicles by type and fuel	Annual	2017	
